# Reviewed article: Amaral AB, Azeredo VMGO, Pereira LA, Rinaldi AEM. Knowledge and actions for the promotion and protection of breastfeeding by primary health care professionals in Uberlândia, 2022. Epidemiol Serv Saude. 2026:35;e20240245.en

**DOI:** 10.1590/S2237-96222026v35e20240245.a

**Published:** 2026-02-16

**Authors:** Joice Ferreira Cunha

**Affiliations:** 1 Hospital Geral de Nova Iguaçu, Nova Iguaçu, RJ, Brazil

## Peer review A

## Questionnaire

Does the manuscript contain new and significant information to justify publication? Yes

Does the Abstract (Summary) clearly and accurately describe the content of the article? Yes

Is the problem significant and concisely stated? Yes

Are the methods described comprehensively? No

Are the interpretations and conclusions justified by the results? No

Is adequate reference made to other work in the field? No

## Manuscript Structure

Length of article is: Adequate

Number of tables is: Too few

Number of figures is: Adequate

Please state any conflict(s) of interest that you have in relation to the review of this paper. None

Interest: Good

Quality: Average

Originality: Good

Overall: Average

Recommendation: Major Revision

## Comments

• Título

Comentário 1: O estudo avaliou as ações e o conhecimento dos profissionais de saúde sobre as práticas de promoção e proteção ao aleitamento materno. Avalie ampliar o título de forma que inclua a população de estudo e o tópico conhecimento. Sugestão: "Conhecimento e ações de promoção e proteção ao aleitamento materno por profissionais da atenção primária a saúde em Uberlândia, Minas Gerais, Brasil". Sugiro remover do título estudo transversal para que o título não fique muito extenso.

• Resumo

Comentário 2: Sugiro incluir a sigla para Norma Brasileira de Comercialização de Alimentos para Lactentes e

Crianças de Primeira Infância (NBCAL), por ser amplamente utilizada na literatura e melhora a fluidez do texto.

Comentário 3: Sugiro da mesma forma, recomendo inserir no texto a sigla para Sistema de Vigilância

Alimentar e Nutricional (SISVAN) para padronização e melhor fluidez.

Comentário 4: Sugiro revisão da metodologia, pois, segundo método descrito, não é adequado afirmar que os resultados foram expressos em prevalência.

Comentário 5: Nas linhas 20 ao 21, recomendo reformular a descrição dos resultados para maior clareza.

Por exemplo, ao apresentar o conhecimento dos coordenadores seria mais claro afirmar que "todos os coordenadores possuíam conhecimento".

• Introdução

Comentário 6: No segundo parágrafo, segunda frase, linha 54, o autor escreve que a norma foi criada como resposta ao código internacional de comercialização de substitutos do leite materno, recomendo substituir o termo como resposta por a partir do. A frase ficaria da seguinte forma: "Esta norma foi criada a partir do

Código....".

Comentário 7: Sugiro revisar a literatura, considerando a importância do conhecimento dos profissionais da saúde sobre aleitamento materna na prática adequada de promoção e proteção do aleitamento materno.

Esses dados podem ser destacados na introdução para justificativa da relevância do tema do estudo.

• Métodos

Comentário 8: Recomendo incluir no primeiro parágrafo da metodologia, página 2, a cobertura da assistência prestada pela atenção básica.

Comentário 9: No terceiro parágrafo da metodologia, o autor declara que o questionário foi aplicado por um único pesquisador. Sugiro descreva se houve revisão do questionário para evitar erro de preenchimento.

Comentário 10: Na página 3, o autor descreve o questionário utilizado, nas seções dois, três e quatro, a redação sugere que as perguntas eram abertas e posteriormente categorizadas para análise. Recomendo maior detalhamento do questionário e do processo de categorização das respostas, pois a conversão de respostas abertas em dados quantitativos poderia introduzir viés e não é adequado estimar prevalência.

Comentário 11: Sugiro descreva o processo de consentimento de participação dos profissionais de saúde e coordenadores para participação do estudo.

• Resultado

Comentário 12: Sugiro incluir a tabela 1 com as características da amostra, para melhor contextualização dos resultados.

Comentário 13: Sugiro revisar a forma de apresentação dos resultados, por exemplo: na linha 14 da página 4, menciona-se que 1% dos profissionais técnicos possuem pós-graduação, essa informação não é essencial pois a categoria não exige pós-graduação; na linha 42 da página 4, a frase "nenhum coordenador e 4,3% dos profissionais...", poderia ser reformulada para "nenhum coordenador e apenas oito profissionais", tornando a informação mais clara.

• Discussão

Comentário 14: Recomendo expandir a discussão com base na literatura, comparando os resultados com os obtidos em estudos semelhantes. Alguns pontos não apresentam comparação com outros trabalhos, como o segundo e terceiro parágrafo da discussão.

Comentário 15: Na página 5, linha 37, sugiro reescrever a frase para maior fluidez e clareza. Sugestão:

"observou uma baixa proporção de profissionais que relataram fornecer orientações ás mães sobre ...".

Comentário 16: Na página 6, segundo parágrafo, o autor menciona a suspensão das atividades durante a pandemia. Entretanto, essa informação não foi detalhada nos métodos. Recomendo revisar e incluir uma descrição mais precisa na metodologia.

Comentário 17: Na página 6, segundo parágrafo, sugiro incluir referência a estudos que discutem o impacto da pandemia no apoio ao aleitamento materno.

Comentário 18: Na página 6, terceiro parágrafo, sugiro expandir a discussão com estudos que abordam

estratégias de promoção e proteção ao aleitamento materno na atenção básica.

